# Alternative Blood Products and Clinical Needs in Transfusion Medicine

**DOI:** 10.1155/2012/639561

**Published:** 2012-04-08

**Authors:** Carolyn Whitsett, Stefania Vaglio, Giuliano Grazzini

**Affiliations:** ^1^Tisch Cancer Institute, Mount Sinai School of Medicine, New York, NY 10029, USA; ^2^Kings County Hospital, Brooklyn, NY 11203, USA; ^3^Centro Nazionale Sangue, Istituto Superiore Sanità, 00161 Rome, Italy

## Abstract

The primary focus of national blood programs is the provision of a safe and adequate blood supply. This goal is dependent on regular voluntary donations and a regulatory infrastructure that establishes and enforces standards for blood safety. Progress in ex vivo expansion of blood cells from cell sources including peripheral blood, cord blood, induced pluripotent stem cells, and human embryonic stem cell lines will likely make alternative transfusion products available for clinical use in the near future. Initially, alloimmunized patients and individuals with rare blood types are most likely to benefit from alternative products. However, in developed nations voluntary blood donations are projected to be inadequate in the future as blood usage by individuals 60 years and older increases. In developing nations economic and political challenges may impede progress in attaining self-sufficiency. Under these circumstances, ex vivo generated red cells may be needed to supplement the general blood supply.

## 1. Introduction

In the last 30 years, transfusion medicine has evolved from a field focused on blood component therapy and red blood cell serology to include advanced cellular therapies produced by ex vivo expansion [1, 2]. The broadest applications of cellular therapies to date have been in the area of regenerative medicine and have involved ex vivo cultured fibroblasts, keratinocytes, and chondrocytes (reviewed in [3]), but substantial progress has also occurred with cellular therapies involving lymphoid and hematopoietic cells. Infusions of donor lymphoid cells to enhance engraftment following allogeneic stem cell transplantation is a common practice [4], and progress has occurred in the development of adoptive T-cell therapies and cellular vaccines [5, 6]. Sipuleucel-T, an autologous cancer vaccine prepared from peripheral blood mononuclear cells (PBMCs) that have been activated and expanded ex vivo with a recombinant fusion protein consisting of prostatic acid phosphatase fused to granulocyte-macrophage colony-stimulating factor (GM-CSF), has been licensed for treatment of castration-resistant prostate cancer [6, 7]. Experimental therapies using ex vivo expanded cells are also being evaluated to improve the results of umbilical cord blood transplantation in adults [8, 9]. Infusion of notch-mediated ex vivo expanded cord blood progenitors shortens the time to neutrophil recovery [8] and infusion of ex vivo expanded T regulatory cells appeared to decrease the severity of acute graft versus host disease [9]. Substantial progress has also occurred in developing culture systems for ex vivo generation of red blood cells [10–17]. The cell sources under evaluation include peripheral blood mononuclear cells (PBMCs), CD 34^pos^ mobilized cells, cord blood (CB), induced pluripotent stem cells (iPSCs) and human embryonic stem cell (hESC) lines [10–16] (Table 1).

The United States Department of Defense is supporting research focused on ex vivo generation of red blood cells from cord blood on an industrial scale. Arteriocyte, a biotechnology company in Cleveland, Ohio, with a coalition of academic and industrial scientists supported in part by research funding from the United States Department of Defense recently provided red cells generated from cord blood to the United States Food and Drug Administration (US FDA) for evaluation [ABC newsletter July 23, 2010]. On March 8, 2011, Cellectis, a French biotechnology company, announced StemRed a joint venture with Etablissement Francais du Sang that will develop red cells ex vivo from induced pluripotent stem (iPS) cells [http://www.dailymarkets.com/stock/2011/03/08/cellectis-and-the-etablissement-francais-du-sang-launch-stemred-a-program-to-produce-red-blood-cells-from-stem-cells/]. These efforts to produce red blood cells in vitro using existing biotechnologies recently led to the first-in-man injection of ex vivo generated red cells in an autologous situation [17].

Scientists studying ex vivo production of platelets have refocused on animal models to clarify the optimal cell product for transfusion, that is, megakaryocytes or mature platelets [18] while efforts continue to improve ex vivo platelet production from umbilical cord blood hematopoietic progenitors using immobilized glycosaminoglycans to enhance megakaryocyte progenitor expansion and platelet release from CD41a^pos^ megakaryocyte progenitors in vitro by inhibiting megakaryocyte apoptosis [19].

Efforts to generate neutrophils are also continuing, perhaps encouraged in part by clinical trials to determine if larger doses of granulocytes collected by apheresis from donors stimulated with dexamethasone and granulocyte colony stimulating factor (G-CSF) would improve clinical outcomes in neutropenic patients with sepsis [20–22]. In this regard it is important to note that a Phase I/II trial sponsored by Cellerant Therapeutics and the Department of Health and Human Services is evaluating the safety, tolerability, and activity of ex vivo expanded human myeloid progenitor cells (CLT-008) administered after up to 5 days of “standard of care” cytarabine-based consolidation chemotherapy in acute lymphoblastic leukemia, acute myeloid leukemia, and high-risk myelodysplasia [http://clinicaltrials.gov/ct2/show/NCT01297543]. Given the enormous effort currently focused on ex vivo expansion of blood cells in general and the progress in ex vivo expansion of red blood cells in particular, the potential clinical benefits to be derived from such alternative products should be considered.

## 2. Materials and Methods

The authors conducted searches on PubMed and Google using the following search terms: adverse reactions, allergic transfusion reactions, alloimmunization, blood group antigens, blood safety, blood supply, blood collection, blood donors, blood transfusion, developing countries, developed countries, emerging diseases, HLA antigens, hemovigilance, history of blood transfusion, neutrophil-specific antigens, platelet-specific antigens, transfusion medicine, transfusion medicine research, clinical trials, transfusion related acute lung injury, and transfusion-transmitted infections. In addition the following websites were consulted: European Medicines Association (EMEA), World Health Organization, United States Department of Health and Human Services Committee on Blood Safety and Availability, Centers for Blood Evaluation and Research, US FDA, Serious Hazards of Transfusion (SHOT). Consensus on needs in transfusion medicine was developed by a process that involved expert opinion as presented in the scientific literature, position papers and statements by governmental and non-governmental organizations involved in areas of blood safety and availability, and discussions among the authors.

## 3. Results

### 3.1. Perspective on Needs in Transfusion Medicine

Since the beginning of modern transfusion therapy extraordinary efforts have been required to maintain a safe and adequate blood supply [23–25]. Governments have acknowledged the importance of these efforts to the overall public health by supporting the development of national blood programs and creating administrative and regulatory structures to assure the health of blood donors and the safety of blood products [26–28]. The goal for nations has been to attain national self-sufficiency, a target that may be unobtainable for some nations with the current economic, political, and social challenges. For more than a decade efforts to develop national blood programs were supported by the World Health Organization (WHO) Global Collaboration on Blood Safety, a voluntary partnership of internationally recognized organizations with expertise in various aspects of transfusion medicine [29]. WHO emphasized five important strategies considered critical to the development of effective national blood programs. These were recently reiterated in a forum addressing the role of regulatory agencies [27]: (1) “establishment of well-organized, nationally coordinated blood transfusion services with legislation and effective mechanisms for oversight, to ensure the timely availability of safe blood and blood products for all patients requiring transfusion,” (2) “collection of blood from voluntary non-remunerated blood donors from low risk populations,” (3) “performing testing for (a) transfusion-transmissible diseases [including HIV, hepatitis B, hepatitis C, and syphilis] (b) major blood groups and (c) blood compatibility,” (4) “safe and appropriate use of blood and a reduction in unnecessary transfusions,” and (5) “quality systems covering the entire transfusion process, from donor recruitment to the follow-up of the recipients of transfusion.” Alignment of a safe and adequate blood supply with achievement of specific WHO Millennium Development Goals (MDGs) assisted developing nations in establishing priorities [30, 31]. The blood supply impacts MDG 4 reduction of the underfive child mortality rate by two-thirds, MDG5 reduction in the maternal mortality rate by three-quarters, MDG 6A halting and reversing the spread of HIV by assuring that younger individuals have comprehensive correct information about HIV and HIV prevention obtained at school and MDG6C decreasing death rates associated with malaria, often caused by severe anemia. The global economic crisis has required that WHO reassess priorities, and The International Society for Blood Transfusion (ISBT) has established the ISBT Working Party on Global Blood Safety to continue the work begun by the WHO [32].

Adverse reactions following blood transfusion that could not have been predicted based on current scientific knowledge have been a concern throughout the history of transfusion medicine [23–25]. Prevention of transfusion-transmitted infections [33–38] and characterization of human blood groups to assure transfusion of compatible blood products [39–43] have always been priorities, but adverse reactions following transfusion continue to occur despite scientific and technical advances and regulatory harmonization [44–50]. Risks posed by new and emerging infections increase [51–53], and evolving scientific knowledge about the immunologic and physiologic consequences of transfusion [54–59] make the descriptors safe and adequate moving targets.

### 3.2. The Blood Supply

In developed nations the blood supply is adequate overall [60–62], but extreme weather conditions and natural disasters such as the recent earthquake and tsunami in Japan and flooding in the United States disrupt routine blood collection activities leading to temporary shortages. The WHO 2008 Blood Safety Survey, which included data from 164 nations representing 92% of the global population, reported on 91.8 million donations. Forty-eight percent of donations were collected in high-income countries representing only 15% of the world population [63]. The accepted standard for evaluating blood availability is blood donations per 1000-population. In this report the donation rate for high-income countries was on average 36.4 (range 13.3–64.6) donations/1000 population, but the rate was lower in middle income and low income nations: 11.6 (range 1.65–36.2)/1000 population and 2.8 (range 0.40–8.2)/1000 population on average, respectively. While there have been substantial improvements in blood safety as developing countries have implemented the WHO recommended testing for syphilis, hepatitis B, hepatitis C, and human immune deficiency virus (HIV), some countries continue to rely on family/replacement and paid donors (36% low income and 27% middle income, resp.) whereas high-income countries reported only 0.3% of such donors.

The 2009 National Blood Collection and Utilization Survey Report provided information on blood collection and utilization in the United States for 2008 [61]. The whole blood/red cell collection rate per thousand population was 85.8, and 14,855,000 units of red cells were transfused for a transfusion rate of 48.8 units/1000 population. Benchmarking data presented at a 2009 meeting of the Alliance of Blood Operators indicated that for 2007/2008 the transfusion rate was on average lower in the European Union (40/1000 on average, with rates in Germany and Denmark closer to United States) and in Canada (32/1000) compared to the United States (48-49/1000 population) [62]. The average transfused dose of platelets was 5.8/1000 for the US, 4.3 for the National Health Service Blood and Transplant, and ≦4/1000 for France. Plasma supplies also appear to be adequate although shortages of AB plasma and plasma from IgA-deficient donors are occasionally reported to be in short supply. In 2006, 4,010,000 units of fresh frozen and apheresis plasma were transfused in the United States with the median volume of plasma for a single episode being 300 mL. Plasma is also collected for fractionation. There is growing concern among patient advocacy groups that the amount of plasma available for fractionation to produce coagulation factor concentrate, intravenous immune globulin, and other plasma products for rare diseases will be inadequate to meet clinical needs. Most nations permit only non-remunerated blood and plasma collections whereas others (including the United States) permit remunerated as well as non-remunerated plasma donations. Patient advocacy groups are concerned that efforts to collect needed plasma from paid donors may compromise donor standards and disrupt routine blood collections. The issue of paid plasma donation is reviewed elsewhere [64]. The Dublin Consensus Statement summarizes these concerns [65]. Recombinant products have been developed to replace some but not all therapeutic plasma proteins. Movement of source plasma and recovered plasma across borders is quite common and perhaps serves as an example of how blood reserves could be shared across borders with standardized regulatory and manufacturing standards. While these data indicate that the blood supply is adequate in developed countries at the present time, there is growing concern that in the future the blood supply may become inadequate [66–74].

 Several factors are responsible for these concerns. Firstly, the number of conditions for which donors are temporarily or permanently deferred is increasing [48, 51–53, 64, 66]. Globalization and emerging infections are implicated in many of these deferrals [33]. A study that estimated donor eligibility in the US population using 31 exclusionary factors corresponding to AABB standards concluded that conventional methods overestimate eligible donor prevalence by approximately 59% [67]. In addition the aging of the population is expected to impact blood availability by increasing blood usage [68–73]. The world population, currently estimated at 6.8 billion, is projected to reach 7 billion in 2012 and surpass 9 billion by 2050 [74]. Most of the population increase (2.3 billion) will occur in developing countries where individuals 15–59 years old will account for 1.2 billion and individuals 60 years and over will account for 1.1 billion of the expected increase. The population in developed countries is expected to increase from 1.23 billion to 1.28 billion. However, the worldwide population of individuals of 65 years and over is projected by the US Census bureau to increase from 7.7% (2010) to 16.5% in 2050. Blood donations will also be affected by aging of the population [68–80].

 The American Red Cross reported that donations by repeat donors 50 years or older increased from 22.1% of total donations in 1996 to 34.5% of total donations in 2005 [80]. During the same period a decrease in the numbers of male and female donors between 20 and 49 years of age was observed. Data from Germany reflects similar donation patterns in that repeat donors are more likely to belong to older age groups (35–44 years and 45–54 years) [70, 71]. The issue of an aging donor base has been resolved in part by countries such as the United States, the United Kingdom, and Australia by eliminating the upper age limit of 70 and allowing younger donors (16 years) to donate with parental permission [75–79]. The Club 25 concept originally developed in Zimbabwe [79] is being adapted for use in developed countries. Despite these efforts, increased blood usage by older populations remains an issue.

Most transfusions in older individuals are related to diagnoses of cardiovascular disease, cancer, and the need for orthopedic surgery [68, 69]. Investigators from the Finnish Red Cross observed that many developed nations had similar age-standardized mortality rates for cancer, cardiovascular disease, injuries, and noncommunicable disease, and using these data simulated red cell usage per 1000 population between 2010 and 2050 for several developed countries based on age-distributed variation in blood usage in Finland between 2002 and 2006 [72]. The simulation predicted substantial increases in blood usage in developed countries associated with a decrease in the size of the population eligible to donate. Earlier studies had predicted a shortfall in blood collections in the United States [68]. Independent assessments using alternative data sources confirm the projected increase in demand for blood caused by increases in the population of individuals over 60 years of age [71, 73].

### 3.3. Prevention of Transfusion-Transmitted Infections: Global Perspective

Progress in preventing transfusion-transmitted infections was recently reviewed [35] Implementation of restrictive donor eligibility criteria and use of volunteer non-remunerated donors, along with specific testing for syphilis and hepatitis B, hepatitis C, HIV1/2, and HTLV1/2, are critical steps in assuring blood safety. In developing countries recruitment of volunteer non-remunerated donors is impacted by cultural definitions of community [81–83] instead of geographic proximity. As a result, efforts to recruit volunteer donors in some nations have been ineffective. Research conducted on the impact on blood safety of converting family/replacement donors to allogeneic donors in South Africa has demonstrated that when such donors are repeat donors, the blood provided is safer than first-time volunteer donors [84–86]. These observations are consistent with previous studies in developed countries indicating a lower risk of infection for repeat donors and should encourage more innovative approaches for donor recruitment in Sub-Saharan Africa.

The most recent published data from the American Red Cross (using NAT testing) indicate that the residual risk per donated unit is 1 : 1,149,000 for HCV, 1 : 357,000 to 1 : 280,000 for HBV, and 1 : 1,467,000 for HIV [36]. By contrast, the estimated residual risk of transfusion-transmitted HIV in a multinational collaborative study involving five nations in Sub-Saharan Africa which used either an antibody assay or combined p 24antigen/antibody assays reported a residual risk for HIV of 34.1 per 1 million donations which represents 1 in 29,000 donations [86]. In addition to these traditional transfusion transmissible diseases for which all donor blood should be tested, donors are also screened for other diseases based on the geographic location, such as for malaria (either by history or using antibody tests [87, 88]) and for local viral and parasitic diseases such as Chagas' disease [89, 90], dengue [91, 92], West Nile virus, [93–95] and chikungunya [96]. In addition, epidemic viral diseases such as H1N1 and yellow fever must also be considered.

Asymptomatic healthy blood donors may also transmit common viral infections which do not cause harm to the average transfusion recipient but which may represent significant risks in selected patient populations. For example, latent cytomegalovirus infection (CMV) is common in normal donors. Depending on the geographic location and socioeconomic status of donors, 70–100% of donors may be seropositive. Pregnant women, neonates, and other immuno-compromised CMV-negative patients may develop serious infectious complications due to transfusion-transmitted CMV. Currently, leukoreduced blood products and products from CMV seronegative donors are used for populations vulnerable to CMV [97–101]. A meta-analysis comparing leukoreduction and antibody screening as CMV prevention strategies reported that both techniques were effective in reducing transmission but indicated that antibody screening may be more effective [98]. Studies of the natural course of CMV infection in blood donors indicate that CMV DNA may persist up to 269 days following infection [97, 99] and suggest that blood donors who have recently seroconverted be deferred for a year.

Parvovirus B19, an erythrotropic virus that uses the P blood group antigen to infect erythroid progenitors, may cause severe anemia, red cell aplasia, and congenital anomalies when transmitted by transfusion [102–107]. Neonates, pregnant women, and patients with congenital and acquired forms of hemolytic anemia are most vulnerable to this infection, but routine screening of blood donors for parvovirus B19 is not performed. However, plasma pools for fractionation are screened for parvovirus B19 because current fractionation/inactivation techniques do not completely inactivate the virus and transmission of parvovirus by coagulation factor concentrate is well documented. Although the risk of transmission of parvovirus B19 appears low in transfusion recipients because of preexisting protective antibody from past natural infections in many transfusion recipients and blood donors, transmission of parvovirus B19 by transfusion has been recently documented [103–105]. Data from the National Heart Lung and Blood Institute Retrovirus Epidemiology Donor Study-II which tested pre- and posttransfusion samples found no transmission to 24 susceptible recipients from transfusion of components with parvovirus B19 DNA containing less than 10^6^ IU/mL [102]. Data from the Japanese Red Cross hemovigilance system provide an alternative perspective which suggests that specially screened products may be needed for selected populations [104]. Between 1999 and 2008, when parvovirus B19 donor screening was conducted using a cutoff similar to that of the REDS study, eight patients with transfusion transmitted parvovirus B19 DNA were identified and sequence identity between patient and the linked donor was confirmed in 5 cases. Red cell aplasia developed in 3 of the 5 confirmed cases. This report suggests that alternative blood products with lower risk of parvovirus B19 infection should be provided for at-risk patient populations.

### 3.4. Alloimmunization

As the scientific basis for producing better and safer products for transfusion through donor screening and product testing has made transfusion safer, alloimmunization resulting from transfusion has emerged as a major issue in clinical care. The magnitude of this problem led to a recent NHLBI sponsored conference to identify areas in which additional research was needed to better understand factors contributing to alloimmunization as well as research on prevention and management of alloimmunization [108]. Alloimmunization in females may result from exposure to paternal antigens during pregnancy. In the general population alloimmunization occurs following transfusion, solid organ or tissue transplantation, or following hematopoietic stem cell transplantation with partially matched donors.

Blood contains many cells and proteins expressing polymorphic antigen systems that may lead to alloimmunization. Erythrocytes alone may express antigens from 30 blood group systems for which over 300 different antigens have been identified [40–43, 109–112]. Small numbers of residual red cells and leukocytes may contaminate plasma and platelet products causing alloimmunization (anti-D developing following transfusion of Rh positive plasma to Rh negative individuals or anti-HLA following platelet transfusion). Typically blood products are matched for ABO group and Rh (D) only unless patients are alloimmunized. Once alloimmunization occurs subsequent transfusions must be matched unless the antibody is considered clinically insignificant [112]. It is estimated that 1–3% of the general population is immunized to blood group antigens.

Alloimmunization is more common in chronically transfused patients such as patients with hemoglobinopathies or transfusion-dependent myelodysplasia [113–116]. Alloimmunization also occurs more often in communities where blood donors and blood recipients are from different ethnic groups [112]. Investigators observed that the rate of alloimmunization in sickle cell disease (SCD) patients in Jamaica where more blood donors were of African ancestry was lower than the alloimmunization rate of SCD patients in the UK where most blood donors were caucasian. Historically, twenty- to thirty-five percent of chronically transfused patients with sickle cell disease or thalassemia are immunized to blood group antigens [113–116]. When these patients experience a hemolytic transfusion reaction, autoantibodies as well as alloantibodies to transfused cells may be produced, causing a syndrome referred to as hyperhemolysis [117–121]. Limited matching strategies such as matching for Kell, Rh (Cc, D, E, e), Fy, and Jk have reduced overall alloimmunization rates in most chronically transfused patient populations [113, 114] but have led to alloimmunization to variant blood group antigen differences which testing with serological reagents does not identify [122–125]. For example, in the Blue Tag program at Children's Hospital in Philadelphia, established to identify African-American donors antigen-matched for African American patients with SCD, alloimmunization to other more subtle antigen differences such as D-deletion variants developed requiring more precise matching with DNA-based technology [122]. Similar observations have been made in other sickle cell patient populations [123]. Patients with myelodysplasia and myeloproliferative neoplasms may also require chronic transfusion support and become alloimmunized. DNA-based methods are now being used to screen large numbers of blood donors to identify compatible blood for alloimmunized patients [126–137]. In the United States, these methods are not FDA approved and phenotypes are confirmed with serological reagents. While most of this work is occurring at blood centers, a recent study suggests that transfusion service laboratories may be able to identify some units in their existing inventory [138]. Identifying matched blood for patients alloimmunized to multiple blood group antigens and patients with rare blood types (variably defined as 1/1000 or 1/10,000 or fewer compatible donors) is a challenge. Although reference laboratories around the world cooperate in this effort [132], blood for such patients is often in short supply. Blood centers are now using information about rare blood types and the populations most likely to contain compatible donors to develop strategies to recruit such donors. For example, Life Share Blood Centers (Louisiana, USA) has published on its website statistics about the ethnicity of donors testing negative for the blood group antigens S, s, and U (a high-incidence antigen), a phenotype more common in African-Americans than Caucasians. Publication of this data has increased minority participation in blood donation. New York Blood Center has a similar program to recruit donors called Precise-Match, tailored for outreach to the various ethnic groups in New York City.

Platelets express A and B blood group antigens, class I HLA-A and HLA-B locus antigens (HLA-C locus antigens are not expressed well on platelets), and platelet-specific antigens 27 of which are well characterized [139–141]. Antibodies to HLA-A and HLA-B locus antigens are most often implicated in platelet refractoriness, but antibodies to platelet-specific antigens are also implicated [142, 143]. Neonatal thrombocytopenia usually involves platelet-specific antigens (PLA 1–17), but several cases implicating antibodies to HLA antigens have been described. Class I and or class II HLA antigens are also expressed on various lymphocyte and monocyte populations, and granulocytes express unique polymorphic antigens [144, 145]. Plasma proteins also express polymorphisms, but serious transfusion reactions have been documented most often in IgA-deficient and haptoglobin-deficient individuals who develop class-specific antibodies to IgA and haptoglobin, respectively [146, 147]. Immunization to HLA antigens affects not only survival of transfused platelets but also prevents engraftment of hematopoietic stem cells and may lead to allograft rejection of solid organs as well as hematopoietic stem cells. Until recently only alloimmunization of transfusion recipients was thought to be a problem, but recently alloantibodies to HLA and granulocyte antigens in donor plasma (and apheresis platelets) have been documented to be the cause of transfusion-related acute lung injury (TRALI) [140, 148]. Because women are more likely to be alloimmunized, TRALI mitigation strategies include use of male donors, identification of never pregnant female donors, and identification of female donors testing negative for anti-HLA antibodies. Implementation of this strategy has substantially reduced morbidity and mortality from TRALI in developed countries but has once again reduced the numbers of individuals available to donate certain blood components.

Platelet transfusions have steadily increased over the past eight years in developed countries. Transfusion of ABO identical platelet products is preferred, but such products are not always available. Platelet recovery in ABO-incompatible transfusions may be slightly lower, but platelet survival is normal. However, transfusion of ABO-nonidentical platelets has caused serious hemolytic transfusion reactions in some patients because of high-titer anti-A and anti-B in donor plasma [149–153]. While hospitals have policies to manage such transfusions, blood centers have not routinely performed anti-A and anti-B titers to identify high-risk group O donors. In Europe, blood centers are testing products and restricting use to group O recipients (reviewed in [154]). The use of platelet additive solutions on a large scale could also lower the risk of hemolysis because such products contain less plasma. A recent publication reported that de novo HLA allosensitization in patients on ventricular assist devices is lower when patients receive leukoreduced ABO identical products, suggesting that even small amounts of hemolysis might facilitate alloimmunization to HLA [151]. Thus matching both for ABO and HLA may be important in providing transfusion support for highly alloimmunized patients [155].

Refractoriness to platelet transfusion is usually caused by alloimmunization to HLA antigens [142, 143, 155, 156]. The TRAP study, a large randomized clinical trial designed to compare leukoreduction and UVB irradiation in preventing refractoriness to platelet transfusions related to alloimmunization to HLA antigens, reported that 45% of AML patients receiving control platelets but only 18% of patients receiving leukoreduced and 21% of patients receiving UVB-treated platelets were alloimmunized [156]. Following this study, leukoreduced platelet products became the standard of care even though UVB irradiation was also effective. Followup of patients who became alloimmunized to HLA revealed that 56% of patients subsequently became antibody negative [157]. Since UVB irradiation and riboflavin are incorporated in current pathogen inactivation methods for platelets, licensed in Europe but not yet in the US, there will likely be renewed interest in this method to prevent alloimmunization [158]. However, a meta-analysis of randomized controlled trials of pathogen-reduced platelets indicates that posttransfusion-corrected count increments were lower, an observation that will affect dosing strategies [159].

Granulocytes play a crucial role in protecting individuals from a number of pathogens. In the early 1970s, the first studies suggesting that granulocyte transfusions might decrease mortality associated with bacterial infections were published [160]. However, collection of adequate numbers of granulocytes and provision of these products to patients in a timely way is a logistical challenge. The introduction of many new and more effective antibiotics and antifungal agents as well as the routine administration of G-CSF and GM-CSF has substantially reduced mortality from infections. However, considerable mortality and morbidity still occur in neutropenic patients following hematopoietic stem cell transplantation. Clinical trials using granulocyte transfusions have provided mixed results [161]. Currently granulocytes are collected by apheresis from prescreened donors (often regular plateletpheresis donors) using dexamethasone or a combination of dexamethasone and G-CSF and transfused as soon as possible but never more than 24 hours after collection. The logistics of collection, variable yields, and limited information on the best conditions for storage have contributed to the variable outcomes in clinical studies published to date. Alloimmunization to granulocyte-specific antigens appears to inhibit the function of transfused granulocytes [160]. Three clinical centers have recently published retrospective analyses of their experience with granulocyte transfusions using optimal mobilization techniques and reported improved survival [154, 162, 163]. An NHLBI-sponsored phase III clinical trial examining the effectiveness of granulocyte transfusions in individuals with neutropenia and infection following dose-intensive chemotherapy or hematopoietic stem cell transplantation is underway [164]. Should this trial provide support for the effectiveness of granulocyte transfusions, a less labor-intense and more cost-efficient method to produce granulocytes would be desirable.

### 3.5. The Red Cell Storage Lesion

Since the publication in 2008 by Koch et al. [55] of a retrospective study reporting increased risk of postoperative complications and reduced short-term and long-term survival in cardiac surgery patients receiving blood stored for more than two weeks, there has been considerable debate about the benefits of transfusing fresh versus older units of blood [57, 59, 165]. Biochemical changes during red cell storage in currently licensed additive solutions are well documented. However, the primary criteria for licensure are hemolysis (below 0.8% in EU and 1% in the US) and red cell survival not less than 75% at the end of storage [165, 166]. In an editorial discussing the potential clinical relevance of this low level of hemolysis on transfusion recipients, potential differences in the effects of free hemoglobin compared to hemoglobin in microvesicles are discussed referencing studies of thrombin generation by red cell supernatant [166]. It is not currently known if clinical outcomes are affected by the age of blood transfused. However, a retrospective cohort study of individuals in Sweden and Denmark transfused between 1995 and 2002 as recorded in the Scandinavian Donations and Transfusions (SCANDAT) Database which analysed 404,959 transfusion episodes revealed a small (5%) excess mortality for recipients of blood stored for 30–42 days and mixed-age compared to recipients of blood stored for 10–19 days [167]. The authors noted no dose response pattern or effect of leukoreduction and concluded that the differences observed were “compatible with a higher baseline risk among recipients of the very oldest units than with an actual deleterious effect of the oldest units.” However, should either clinical or basic research studies provide conclusive evidence that inferior outcomes are associated with transfusion of blood stored for longer periods, the shift toward usage of fresher blood would reduce blood inventories and create significant blood shortages.

The editorial by Simone A. Glynn, MD, MPH from the Division of Blood Diseases and Resources at the National Heart Lung and Blood Institute summarizes the situation well by stating that, despite the numerous publications, there is “genuine uncertainty as to whether transfusing fresher blood is more, less, or equally beneficial as transfusion of older blood” [59]. The editorial identifies four large randomized clinical trials related to the age of transfused blood that are underway in North America. The Canadian Institutes of Health Research is funding two trials, one in intensive care patients randomized to receive either less than 8-day or standard issue red blood cells (2–42 days) in The Age of Blood Evaluation (ABLE) Study which has as the primary outcome 90-day all-cause mortality and a second study in which premature infants (≤1250 g) will be randomized to receive either less than 8-day or 2–42-day aliquots with a primary endpoint being a 90-day composite measure of all cause mortality and organ dysfunction. The Cleveland Clinic is conducting the Red Cell Storage and Outcomes in Cardiac Surgery Trial (NCT00458783) in which individuals 18 yrs and older undergoing cardiopulmonary bypass for primary and reoperative coronary artery bypass grafting, coronary artery bypass grafting with a valve procedure, and isolated valve procedures are randomized to receive blood transfusion with storage duration less than 14 days or greater than 20 days. NHLBI is also sponsoring through the Transfusion Medicine Clinical Trials Group the Red Cell Storage and Duration Study (RECESS)—NCT00991341 in which individuals 12 years or older undergoing complex cardiac surgery and likely to need transfusion are randomized to receive blood ≤10 days storage duration or ≥21 days storage duration. The primary outcome is the change in the composite multiple organ dysfunction score (MODS). Dr. Glynn also identified research projects funded under the NHLBI program “Immunomodulatory, Inflammatory, and Vasoregulatory Properties of Transfused Red Blood Cell Units as a Function of Preparation and Storage” designed to provide information on mechanisms via which transfusion of older blood might lead to adverse physiological outcomes and to design interventions to eliminate them. Current data are inadequate to determine if transfusion of older units of blood is associated with either short- or long-term adverse outcomes. Basic research in animal models and prospective randomized clinical trials are needed in this area. 

### 3.6. Impact of Ex Vivo Generated Blood Cells on Needs in Transfusion Medicine

 Red blood cells have been successfully generated ex vivo from peripheral blood mononuclear cells, cord blood, and human-induced pluripotent stem cell (hiPSC) and human embryonic stem cell (hESC) lines. The expansion potential of cord blood and peripheral blood is limited, but that of hiPSC and hESC is infinite. Multiple products from a single collection (blood or skin fibroblasts) would be generated from well-characterized donors under GMP conditions. Irrespective of the cell source, the donor will be selected to be negative for known transmissible diseases and will represent either a rare phenotype (1/1000 or ≤1/10,000) or a phenotype much in demand (O Rh negative or O Rh positive). The primary product will be almost devoid of other contaminating blood cells and suspended in defined media designed to support viability and cell function but lacking immunoglobulins and other plasma components which precipitate TRALI and allergic transfusion reactions (IgA, haptoglobin). Theoretically, the equivalent of 10–50 therapeutic units of blood (2 × 10^12^  cells) can be generated from a single cord blood unit. The development of cord blood banks for hematopoietic stem cell transplantation has created an infrastructure that facilitates collection of cord blood for other uses (i.e., endothelial cells for regenerative medicine) [168]. Cord blood can also be cryopreserved for up to 23 years with recovery of adequate numbers of hematopoietic progenitors and the ability to generate iPS [169]. Although the expansion potential of peripheral blood is considered to be lower than that of cord blood, at present peripheral blood is more readily obtained because it is a byproduct of leukoreduction.

The first in-man transfusion is likely to be from one of these two cell sources with studies of red cells generated from hESC or hiPSC coming later [170–172]. Currently 1200 hESC lines have been established worldwide, and 375 of these are deposited in two international registries [172]. Investigators at the forefront of ex vivo expansion studies expect that GMP compliant facilities to produce red cells ex vivo will be available within the next 2-3 years. Location of manufacturing facilities will be critical given the complexity of the manufacturing process and the need for reliable transport. The potential impact of such products on blood manufacturing operations is substantial.

The first clinical use of ex vivo generated red cells is likely to be for highly alloimmunized patients and for patients with rare blood types [170, 171]. Theoretical calculations to determine how many iPSC lines would be needed to support alloimmunized patients in France have already been performed [171]. Although a large number of embryonic stem cell lines have been developed, the expression of blood group antigens by erythrocytes from these cell lines is not known [172]. Forty percent of rare units in France were provided to patients with sickle cell anemia [171]. Data from WHO suggests that worldwide patients with hemoglobinopathies are represented among patients requiring chronic transfusion. Hemoglobinopathies occur in over 332,000 conceptions or births annually and account for 3.4% of under-5 mortality [173, 174]. Worldwide an estimated 275,000 have a sickle cell disorder and 56,000 a major thalassaemia, approximately 30,000 of which will require chronic transfusion to survive. In the WHO America region, 52.4% of transfusion-dependent patients receive transfusions, but in the Eastern Mediterranean region and South-East Asian region, which have a higher number of transfusion-dependent patients, 17.8% and 9.6%, respectively, of transfusion-dependent patients received transfusions [174]. These data suggest that, as healthcare improves in developing nations, the number of patients with hemoglobinopathies requiring transfusions will increase and these individuals will likely need special antigen-matched products.

 Once manufacturing facilities are established for ex vivo generation of red cells, the general inventory of blood in both developing and developed countries could be increased by ex vivo generation of red cells. Theoretically, if peripheral blood mononucleated cells (PBMCs) from each whole blood donation were to be expanded ex vivo, each donation would produce not only one but possibly 3–10 or more units of blood. This would allow blood donors to donate less often, decrease concerns about iron deficiency, and reduce costs for donor recruitment. PBMC generated by leukoreduction during the manufacturing process for red cells are the easiest product to obtain. The least expensive way to prepare leukoreduced products is the buffy coat method, but recovery of PBMC from in-line or sterile docked filters is also possible. In 2006, prestorage leukoreduction was performed on approximately 11.3 million units of red blood cells in the United States. Currently, cord blood units are being collected as a source of hematopoietic progenitor cells for transplantation, but cord blood contains other cell populations that have value in regenerative medicine such as naïve immune cells, mesenchymal cells, and endothelial progenitor cells. Accurate figures for donations to public banks are available. Donation rates to public banks vary from 0.1% of births to 0.34% of births [168]. Some families choose to bank cord blood for autologous/family use, and these units are not available for ex vivo generation of blood cells. Overall, these data indicate that a potentially vast but untapped resource is available for ex vivo generation of blood cells. At present there is no data to suggest that ex vivo products are needed in developed nations for the general inventory but should projections about increased blood usage in individuals age 60 years and older be correct such products may be needed in the future.

The supply of safe blood in developing countries has improved in recent years but is still inadequate. Anemia related to trauma, pregnancy, or malaria is the most common indication for transfusion, and currently blood is not always available when needed. It is unrealistic to propose that a country that cannot effectively run a national blood program would have the resources to expand blood cells ex vivo. However, a manufactured blood product produced elsewhere could be imported. Manufactured products are more uniform because methods and product specifications can be standardized with quality monitoring implemented to assure that such standards are met. Under these circumstances, cellular products might move freely across national borders as is the case with some pharmaceuticals. Alternatively, one might maintain the current not-for-profit business model for whole blood collection and model ex vivo expansion on a for-profit model where economies of scale might work to reduce overall cost.

Progress in ex vivo generation of megakaryocytes and platelets is not as advanced as development of red cells. The value of platelet transfusions in preventing hemorrhage in thrombocytopenic patients and patients with thrombocythemia is not disputed although the level at which platelet transfusions should be given prophylactically to thrombocytopenic patients to prevent hemorrhage was the subject of recent clinical trials. Platelets may be manufactured from whole blood by centrifugation to produce platelet concentrate or prepared by apheresis. An adult dose of platelets would require pooling of 4–6 units of platelets depending on local practice or a single apheresis product. Both products are usually leukoreduced. There is ongoing concern about the adequacy of platelet inventories for two reasons, the shelf life is short (maximum of 5 days) and platelet products stored at room temperature are more likely to be contaminated with bacteria [37, 38]. Refractoriness to platelet transfusions is usually caused by anti-HLA antibodies. Antibodies to platelet-specific antigens may also be implicated. In neonatal thrombocytopenic purpura, the offending antibody is typically directed at platelet-specific antigens. Refractoriness to platelet transfusions is a significant problem for multitransfused patients with hematologic malignancies receiving chemotherapy or undergoing hematopoietic stem cell transplantation. Identification of suitable products for alloimmunized patients involves HLA typing of the patient (typically performed with DNA-based techniques), antibody screening for anti-HLA class I and anti-platelet-specific antibodies (solid phase or flow cytometry) as well as platelet crossmatching (solid phase or flow cytometric techniques). Complex computer algorithms for selecting HLA compatible donors based on epitope sharing have been developed to identify compatible products [142, 143]. Thus the ability to generate platelet products ex vivo and platelet products lacking HLA antigens in serum free media would have great clinical value. Methods have been developed to generate platelets ex vivo from cord blood CD34 positive cells and from embryonic stem cells [175]. Ex vivo generation of platelet products deficient in HLA class I antigen expression could have an enormous impact on the provision of platelet products to refractory patients. Using an RNA-interference-based mechanism (RNAi) in which a lentiviral vector was used to express short-hairpin RNA targeting *β*2-microglobulin transcripts in CD34 positive cells, Figueiredo et al. generated platelets demonstrating an 85% reduction in class I HLA antigens compared with platelets generated in CD34 positive cells transduced with a lentiviral vector containing a nonsense shRNA [175]. These platelets appeared to have normal function in vitro. Alternatively, hESC or hiPSC could be used to generate products for highly immunized patients.

Granulocyte transfusions have not consistently demonstrated improved clinical outcomes in infected neutropenic patients. However, these inconsistent results may reflect heterogeneity in patient populations, failure to transfuse an adequate number of granulocytes, limited information on optimal storage conditions for granulocyte products, and difficulty in identifying a rapid reliable assay for determining granulocyte compatibility (other than ABO). Studies of ex vivo generated neutrophils may provide valuable information on optimal storage conditions for neutrophils and may ultimately produce a product with a longer shelf-life.

 Theoretically, ex vivo generated red blood cells will be produced on demand on a predictable schedule, will provide cellular products of more uniform composition with limited contamination by other cell types, will be associated with lower rates of transmission of infectious diseases, cause fewer allergic reactions, produce a lower rate of alloimmunization because extended matching will be possible, and will have longer in vivo survival while producing less iron overload than currently available products. It is not possible to predict when these products will be approved for clinical use. Many complex issues related to scale-up production and the potential immunogenicity (neoantigen formation) of products produced in vitro remain to be resolved. However, the development of these products will inform and transform quality control and manufacturing processes for traditional blood products.

## Figures and Tables

**Table 1:** Cell sources for ex vivo generation of red blood cells*.

Cell type	Source	Special consent required	Advantages	Disadvantages
Peripheral blood mononuclear cells (PBMCs)	By product of leukoreduction	No	Donor with known phenotype	Limited expansion capacity
	Apheresis collection	Yes	≥3 products from single collection	Donors may become ineligible
	Mobilizing agent before apheresis	Yes (for mobilization and apheresis)	Expansion similar to cord blood with humanized media	Quality control requirement greater than blood
Cord blood	Low volume units unsuitable for transplantation	Yes	Hematopoietic progenitor cell number > PBMC 3–50 products phenotype must be determined	Multiple donations not possible
				Quality control moderately complex
iPSC	Fibroblast cultures from autologous or allogenic donors of known phenotype	Yes (for skin biopsy, fibroblast culture, and induction of pluripotency)	Unlimited expansion capacity	Quality control highly complex
hESC	Human embryos *≃*1200 cell lines available	Yes	Unlimited expansion capacity	Ethical limitations on development and use
				Quality control highly complex

*Bacterial, mycoplasma, fungal, or viral contamination possible.
